# **RETRACTION**: The Sp1/FOXC1/HOTTIP/LATS2/YAP/β‐catenin cascade promotes malignant and metastatic progression of osteosarcoma

**DOI:** 10.1002/1878-0261.13692

**Published:** 2024-07-01

## Abstract

**RETRACTION**: K. Liu, J.‐D. Ni, W.‐Z. Li, B.‐Q. Pan, Y.‐T. Yang, Q. Xia and J. Huang, “The Sp1/FOXC1/HOTTIP/LATS2/YAP/β‐Catenin Cascade Promotes Malignant and Metastatic Progression of Osteosarcoma,” *Molecular Oncology* 14, no. 10 (2020): 2678–2695, https://doi.org/10.1002/1878-0261.12760.

The above article, version of record published online on 29 August 2020 in Wiley Online Library (wileyonlinelibrary.com), has been retracted by agreement between the journal Editor in Chief, Kevin Ryan and John Wiley and Sons Ltd. The decision to retract the manuscript has been made due to insufficient explanation or provision of appropriate raw data to justify the duplicated ruler, which have appeared in other publications, along with overlap between different panels of Figs. 2E, 3F, and 4F. The editors consider the conclusions substantially compromised and are therefore retracting the paper.

**RETRACTION**: K. Liu, J.‐D. Ni, W.‐Z. Li, B.‐Q. Pan, Y.‐T. Yang, Q. Xia and J. Huang, “The Sp1/FOXC1/HOTTIP/LATS2/YAP/β‐Catenin Cascade Promotes Malignant and Metastatic Progression of Osteosarcoma,” *Molecular Oncology* 14, no. 10 (2020): 2678–2695, https://doi.org/10.1002/1878-0261.12760.

The above article, version of record published online on 29 August 2020 in Wiley Online Library (wileyonlinelibrary.com), has been retracted by agreement between the journal Editor in Chief, Kevin Ryan and John Wiley and Sons Ltd. The decision to retract the manuscript has been made due to insufficient explanation or provision of appropriate raw data to justify the duplicated ruler, which have appeared in other publications, along with overlap between different panels of Figs. 2E, 3F, and 4F. The editors consider the conclusions substantially compromised and are therefore retracting the paper.

